# Membrane Targeting of C2GAP1 Enables *Dictyostelium discoideum* to Sense Chemoattractant Gradient at a Higher Concentration Range

**DOI:** 10.3389/fcell.2021.725073

**Published:** 2021-07-30

**Authors:** Xuehua Xu, Smit Bhimani, Henderikus Pots, Xi Wen, Taeck J. Jeon, Arjan Kortholt, Tian Jin

**Affiliations:** ^1^Chemotaxis Signaling Section, Laboratory of Immunogenetics, National Institute of Allergy and Infectious Diseases, National Institutes of Health, Rockville, MD, United States; ^2^Department of Cell Biochemistry, Univeristy of Groningen, Groningen, Netherlands; ^3^Department of Biology and BK21-Plus Research Team for Bioactive Control Technology, College of Natural Sciences, Chosun University, Gwangju, South Korea

**Keywords:** chemotaxis, C2GAP1, adaptation, sensitivity, G protein coupled receptor, gradient sensing

## Abstract

Chemotaxis, which is G protein-coupled receptor (GPCR)-mediated directional cell migration, plays pivotal roles in diverse human diseases, including recruitment of leukocytes to inflammation sites and metastasis of cancer. It is still not fully understood how eukaryotes sense and chemotax in response to chemoattractants with an enormous concentration range. A genetically traceable model organism, *Dictyostelium discoideum*, is the best-studied organism for GPCR-mediated chemotaxis. Recently, we have shown that C2GAP1 controls G protein coupled receptor-mediated Ras adaptation and chemotaxis. Here, we investigated the molecular mechanism and the biological function of C2GAP1 membrane targeting for chemotaxis. We show that calcium and phospholipids on the plasma membrane play critical roles in membrane targeting of C2GAP1. Cells lacking C2GAP1 (*c2gapA*^–^) displayed an improved chemotaxis in response to chemoattractant gradients at subsensitive or low concentrations (<100 nM), while exhibiting impaired chemotaxis in response to gradients at high concentrations (>1 μM). Taken together, our results demonstrate that the membrane targeting of C2GAP1 enables *Dictyostelium* to sense chemoattractant gradients at a higher concentration range. This mechanism is likely an evolutionarily conserved molecular mechanism of Ras regulation in the adaptation and chemotaxis of eukaryotes.

## Introduction

Chemotaxis, which is G protein-coupled receptor (GPCR)-mediated directional cell migration, plays pivotal roles in diverse physiological and pathological processes, such as embryo development, angiogenesis, recruitment of neutrophils to inflammation sites, and metastasis of cancer. *Dictyostelium* is a genetically traceable model organism that is the best-studied organism for GPCR-mediated chemotaxis. Chemotaxis is crucial for detecting, moving toward, and eventually engulfing bacteria as food source, as well as development of *Dictyostelium*. A key feature of chemotaxis in eukaryotic cells is that cells sense a large concentration range of chemoattractants (10^–9^–10^–5^ M cAMP in *Dictyostelium* and 10^–9^–10^–5^ M fMLP in neutrophils). To accurately navigate through an enormous concentration-range gradient of various chemoattractants, both *Dictyostelium* and neutrophils employ a mechanism called adaptation, in which they no longer respond to present stimuli but remain sensitive to stronger stimuli. Homogeneous, sustained chemoattractant stimuli trigger transient, adaptive responses in many steps of the GPCR-mediated signaling pathway downstream of heterotrimeric G proteins (Janetopoulos et al., 2001; Hoeller et al., 2014). Adaptation provides a fundamental strategy for eukaryotic cell chemotaxis through large concentration-range gradients of chemoattractants. However, little connection has been made between GPCR-mediated adaptation and the basal activity of a cell.

The small GTPase Ras mediates multiple signaling pathways that control directional cell migration in both neutrophils and *Dictyostelium* (Zheng et al., 1997; Sasaki et al., 2004; Zhang et al., 2008; Suire et al., 2012; Wang et al., 2014; van Haastert et al., 2017). In *Dictyostelium*, the activation of Ras is the first signaling event that displays adaptation behavior in GPCR-mediated signaling pathways (Sasaki et al., 2004). Ras signaling is activated through guanine nucleotide exchange factors (GEFs) and deactivated by GTPase-activating proteins (GAPs). Several RasGEFs and RasGAPs have been found to activate and deactivate Ras signaling, respectively (Insall et al., 1996; Zhang et al., 2008; Xu et al., 2017). Recently, we identified a locally recruited RasGAP protein, C2GAP1, that is essential for F-actin-independent Ras adaptation and long-range chemotaxis in *Dictyostelium* (Xu et al., 2017). However, the molecular mechanism of membrane targeting of C2GAP1 is not fully understood. Here, we show that calcium and phospholipids on the plasma membrane, but not GAP activity, play critical roles in membrane targeting of C2GAP1. More importantly, C2GAP1 controls both the basal activity and the GPCR-mediated adaptation in *Dictyostelium* cells and thereby enables cells to sense chemoattractant gradients at a higher concentration range.

## Results

### Calcium Negatively Mediates Membrane Targeting of C2GAP1

**FIGURE 1:**
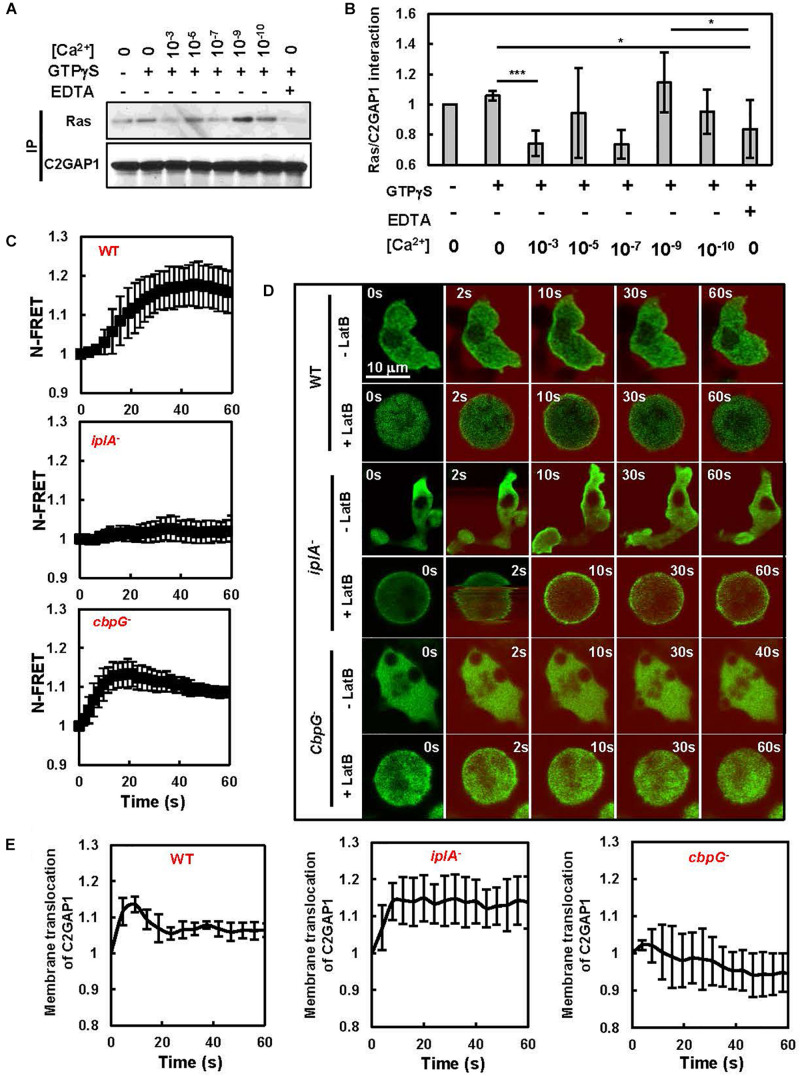
[Ca^2+^] is dynamically involved in the membrane targeting of C2GAP1. **(A)** High [Ca^2+^] decreases the association of C2GAP1-YFP and Ras determined by co-immunoprecipitation (co-IP). Cells expressing C2GAP1-YFP were lysed in the presence or absence of GTPγS (10 μM), CaCl_2_ at the indicated concentrations, or 2 mM EDTA and then subjected to IP assay and western blot detection of the indicated protein with their specific antibodies. **(B)** Quantitative measurement of C2GAP1 and Ras association presented in **(A)**. The ratio of Ras and C2GAP1 at time 0 s was normalized to 1. Mean ± SD from three independent experiments is shown. The *p*-values of Student’s *t*-test are indicated as * (*p* < 0.1) and *** (*p* < 0.001). **(C)** cAMP-induced calcium response in WT, *iplA*^–^, or *cbpG*^–^ cells using calcium FRET sensor YC-Nano15. Cells expressing YC-nano15 were stimulated with 1 μM cAMP at 0 s. To eliminate cell migration for quantitative measurement, cells were treated with 5 μM Lat B for 10 min prior to the experiment. Calcium response was measured by sensitized emission FRET using a Zeiss 780 fluorescent microscope, analyzed using Zen software, and then graphed with Microsoft Excel. The FRET signal (N-FRET) at time 0 s was normalized to 1. Mean ± SD is shown; *n* = 7, 8, or 8 in WT, *iplA^–^*, or *cbpG*^–^ group, respectively. **(D)** Montage shows cAMP-induced membrane translocation of C2GAP1-YFP (green) in WT, *iplA*^–^, or *cbpG*^–^ cells in response to homogeneously applied 10 μM cAMP stimulation (red). cAMP-chemotactic cells expressing C2GAP-YFP (green) were stimulated with cAMP at time 0 s. To visualize the stimuli, cAMP was mixed with fluorescent dye Alexa 594 (red). Also see Supplementary Video 1. **(E)** Quantitative measurement of membrane-bound C2GAP1-YFP upon exposure to a cAMP gradient is shown. The quantitative measurement of membrane translocation was measured from the cells treated with 5 μM Lat B shown in **(D)** Membrane translocation of C2GAP1 was quantified by the decreased in the cytosolic intensity of C2GAP1. The cytosolic intensity at time 0 s was normalized to 1. Mean ± SD is shown; *n* = 5, 6, or 8 for the groups of WT, *iplA*^–^, or *cbpG*^–^ cells, respectively.

C2GAP1 possesses a C2 domain, which is a calcium-binding motif and is often involved in membrane targeting of host proteins (Nalefski and Falke, 1996; Corbalan-Garcia and Gomez-Fernandez, 2014). Thus, we first examined whether calcium affects the interaction between C2GAP1 and Ras by immunoprecipitation and found an interesting effect of [Ca^2+^] on the interaction between C2GAP1 and Ras (Figure 1A). The presence of high [Ca^2+^] (> 100 nM) decreased the C2GAP1/Ras interaction. Interestingly, the highest interaction of C2GAP1 and Ras was detected with the presence of 1 nM calcium, while lower [Ca^2+^] to none again decreased the interaction (Figure 1B). The effect of [Ca^2+^] on the interaction between C2GAP1 and Ras might be due to its effect on C2GAP1 membrane targeting. To understand the effect of [Ca^2+^], we first monitored the cAMP-induced calcium response by an ultrasensitive [Ca^2+^] indicator, Nano15, using fluorescence resonance energy transfer (FRET) microscopy in live cells (Horikawa et al., 2010; Xu et al., 2016). Consistent with a previous report (Horikawa et al., 2010), 1 μM cAMP stimulation induced a transient increase in the FRET efficiency of Nano15, indicating a clear [Ca^2+^] increase peaking around 40 s after stimulation in wild-type (WT) cells (Figure 1C). In *iplA*^–^ cells, which lack the IP_3_ receptor, cAMP stimulation triggered no [Ca^2+^] increase, as previously reported (Traynor et al., 2000). Calcium-binding protein 7 (CbpG) has the highest calcium-binding capacity among 14 *Dictyostelium* calcium-binding proteins (Cbps) and is highly expressed during early development (Sakamoto et al., 2003). Cells lacking Cbp7 (*cbpG*^–^) have a slightly higher level of [Ca^2+^] in the resting state, indicating that Cbp7 functions as a Ca^2+^-binding protein (Wilczynska et al., 2005; Park et al., 2018). In response to 1 μM cAMP stimulation, *cbpG*^–^ cells showed a much more rapid [Ca^2+^] increase with the peak at about 20 s. This [Ca^2+^] dynamics is typically observed in *Dictyostelium* mutants that lack calcium-binding proteins: earlier onset, earlier peak, and faster fall rate (Wilczynska et al., 2005).

We next monitored membrane translocation of C2GAP1-YFP in WT and mutant cells (Figure 1D and Supplementary Video 1). We found that 1 μM cAMP induced a robust membrane translocation of C2GAP1-YFP in WT cells (Supplementary Video 1, top left). The same cAMP stimulation induced a prolonged membrane translocation of C2GAP1 in *iplA*^–^ cells (Supplementary Video 1, middle left). In the resting *cbpG*^–^ cells, membrane localization of C2GAP1-YFP was notably increased. cAMP stimulation triggered little membrane translocation in *cbpG*^–^ cells (Supplementary Video 1, bottom left). We also monitored C2GAP1-YFP in WT, *iplA*^–^, and *cbpG*^–^ cells treated with 5 μM Latrunculin B (+ LatB), an actin polymerization inhibitor, to eliminate cell migration for quantitative measurement of membrane translocation of C2GAP1-YFP. Interestingly, there was notably more membrane localization of C2GAP1 in resting *iplA*^–^ and *cbpG*^–^ cells (Supplementary Figure 1). In WT cells, cAMP stimulation induced membrane translocation of C2GAP1-YFP that was similar in both immobile cells and motile cells (Supplementary Video 1, top right). cAMP stimulation triggered a significantly prolonged membrane translocation of C2GAP1 in *iplA*^–^ cells (Supplementary Video 1, middle right), while the same cAMP stimulation induced little membrane translocation of C2GAP1, instead, further reduced membrane localization of C2GAP1 in *cbpG*^–^ cells (Supplementary Video 1, bottom right). Quantitative measurement of the precise membrane translocation dynamics of C2GAP1 in these cells confirmed what we observed in motile cells (Figure 1E). In contrast to WT cells, cAMP stimulation triggered a significant prolonged membrane translocation of C2GAP1 in *iplA*^–^ cells and little membrane translocation in *cbpG*^–^ cells. Taken together, the above results indicate that calcium dynamically regulates membrane targeting of C2GAP1.

### C2GAP1 Binds to Phospholipids on the Plasma Membrane

**FIGURE 2:**
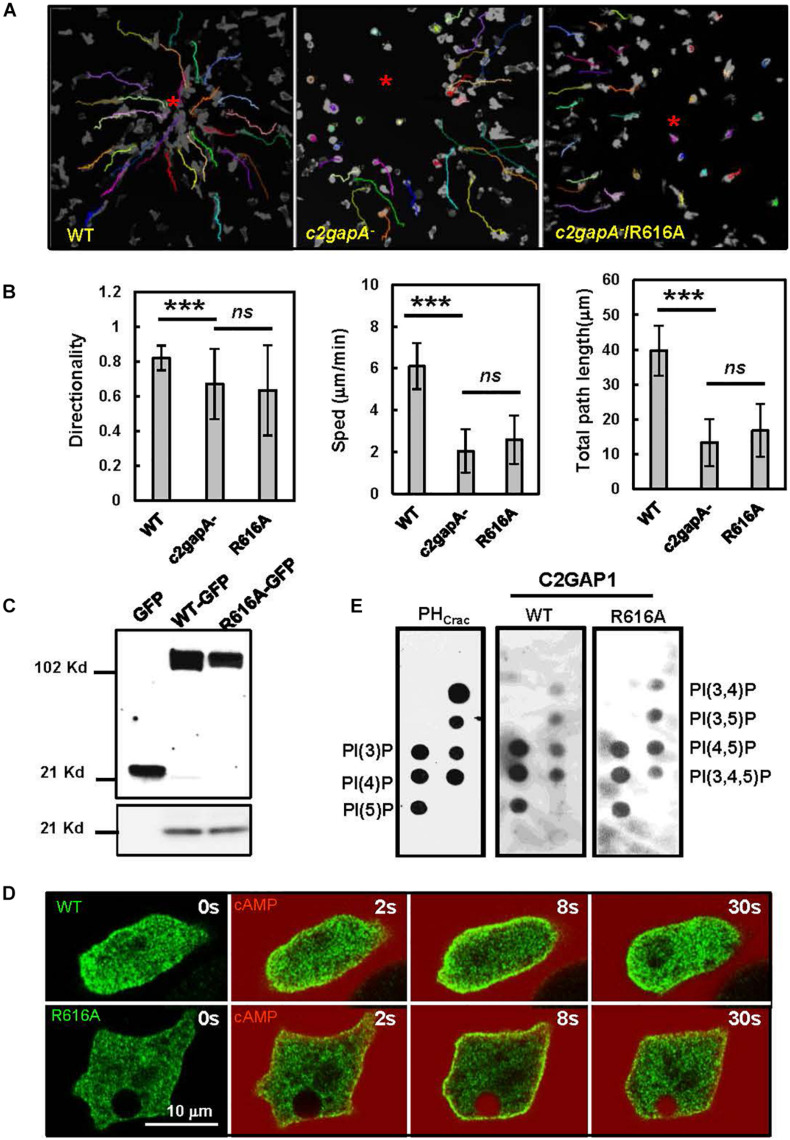
GAP activity is not required for the binding of C2GAP1 and phospholipids on the plasma membrane. **(A)** Montage show the tracing paths of chemotaxing cells of WT or *c2gapA*^–^ cells expressing empty vector (*c2gapA*^–^), or R616R (*c2gapA*/R616A) mutant of C2GAP1. Red dots indicate the source of the cAMP gradients (10 μM). **(B)** Quantitative measurements of chemotaxis parameters such as directionality, speed, and total path length using FIJI(NIH) with the MTrackJ plugin. Directionality, where 0 represents random movement and 1 represents straight movement toward the source of a gradient; speed, defined as the distance that the centroid of the cell moves as a function of time; total path length, the total distance the cell has traveled. 30 cells were traced in each group were measured for 5 min in each group. Mean ± SD is shown. The *p-*values of Student’s *t*-test are indicated as *ns* (not significant, *p* > 0.05) and *** (*p* < 0.001). **(C)** Interaction between WT or R616A mutant of C2GAP1 and Ras determined by co-IP. Lysates of cells expressing GFP or GFP-tagged WT (WT-GFP) or R616A (R616A-GFP) of C2GAP1 were incubated with agarose beads conjugated with anti-GFP antibodies and then subjected to immunoprecipitation and western blot detection of GFP and Ras using their specific antibodies. **(D)** cAMP stimulation triggers membrane translocation of WT or R616A mutant of C2GAP1 in the cells. Cells expressing GFP-tagged WT or R616A (green) were stimulated with 10 μM cAMP. The application of cAMP was visualized by mixing cAMP with a fluorescent dye, Alexa 594 (red). Also see Supplementary Video 2. **(E)** Phospholipid binding of WT or R616A mutant of C2GAP1. Lysates of cells expressing either PH_Crac_-GFP, WT-GFP, or R616A-GFP were incubated with PIP strips and then subjected to western blot detection by anti-GFP. The western blotting of three PIP-strip membranes were performed using the same procedures.

Proteins and phospholipids on the plasma membrane play critical roles in membrane targeting of C2 domain-containing RasGAPs (Corbalan-Garcia and Gomez-Fernandez, 2014; Xu et al., 2017). In order to understand whether GAP activity plays a crucial role in the interaction between Ras and RasGAP1, we generated a GFP-tagged GAP-inactive mutant of C2GAP1 (R616A) and expressed it in *c2gapA*^–^ cells (*c2gapA*^–^/R616A). We then examined the chemotaxis behavior of *c2gapA*^–^/R616A cells and compared it with that of WT and *c2gapA*^–^ cells (Figure 2A). We found that *c2gapA^–^/*R616A cells displayed similar chemotaxis defect to *c2gapA*^–^ cells in a 10 μM cAMP gradient in contrast to WT cells, as previously reported (Figure 2B). The above result indicates that R616A does not rescue the chemotaxis defect of *c2gapA*^–^ cells. Using immunoprecipitation, we found that the R616A mutant showed a binding with Ras similar to that of the WT C2GAP1 (Figure 2C). The above results indicate that GAP activity is not required for the interaction between C2GAP1 and Ras.

Phospholipids on the plasma membrane play critical roles in the membrane targeting of C2 domain-containing proteins (Corbalan-Garcia and Gomez-Fernandez, 2014). We found that cAMP stimulation also triggered clear membrane translocation of R616A (Figure 2D and Supplementary Video 2). Therefore, we used a PIP strip assay to determine the species of phospholipids that bind to C2GAP1. We incubated PIP strips with cell lysates collected from the cells expressing GFP-tagged PH_*C*__*rac*_ (PH_Crac_-GFP), WT, or R616 of C2GAP1. PH_Crac_-GFP was used as a control. The phospholipid-binding specificity of PHCrac-GFP is consistent with the previous report (Zhang et al., 2010). We found that both WT and R616A of C2GAP1 bound to multiple phospholipids (Figure 2E), including the products of PI3K, such as PI(3)P, PI(3,4)P2, and PI(3,4,5)P3, and the substrates of PI3K. Interestingly, R616A showed a preference for PI(4,5)P2, while the WT showed a better affinity to PI(3,4,5)P3.

### Sensitivity of *c2gapA*^–^ Cells Is Increased in Response to Chemoattractant Stimulus

**FIGURE 3:**
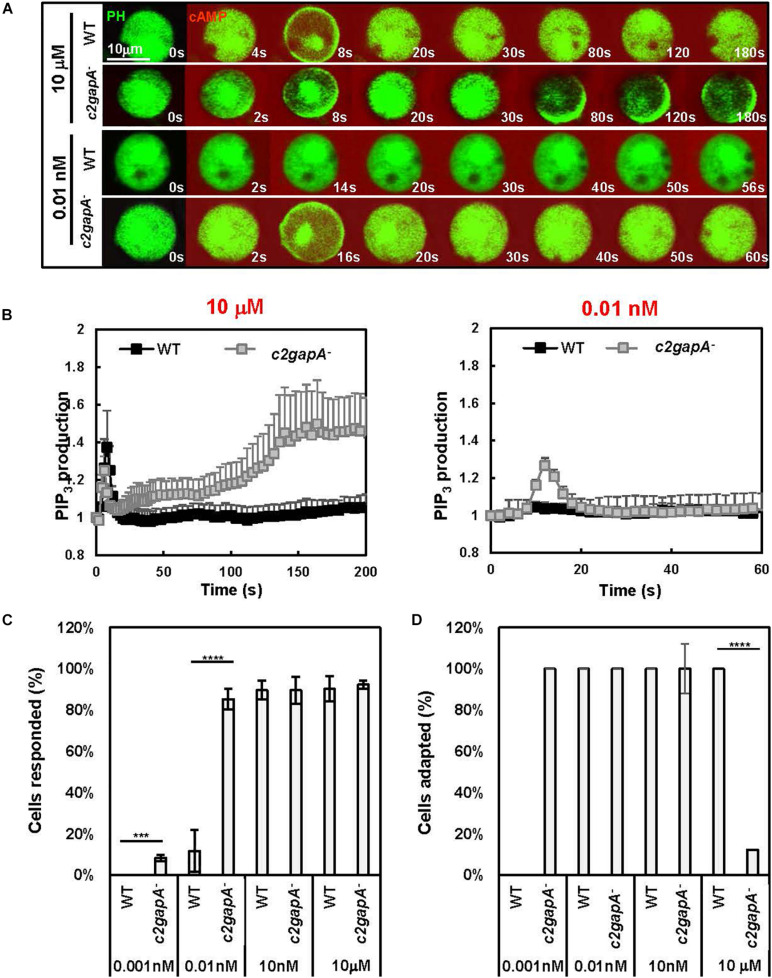
*c2gapA*^–^ cells are more sensitive to the stimuli at low concentrations and fail to adapt to the stimuli at high concentrations. **(A)** Montage shows cell response as PIP_3_ production monitored through the membrane translocation of the PIP_3_ probe PH_Crac_-GFP, in WT or *c2gapA*^–^ cells. Cells were stimulated with either 0.01 nM or 10 μM Sp-cAMPS, respectively. Also see Supplementary Videos 3, 4 for cell response to 10 μM or 0.01 nM Sp-cAMPS stimulation, respectively. **(B)** Quantitative measurement of PIP3 production in WT and *c2gapA*^–^ in response to 10 μM or 0.01 nM Sp-cAMP stimulation shown in **(C)**. PIP3 production was quantified by the decrease in the cytosolic intensity of PH_Crac_-GFP. The cytosolic intensity at time 0 s was normalized to 1. Mean ± SD is shown; *n* = 8 and 12, or 8 and 11 for WT, *c2gapA*^–^ cells in response to 10 μM or 0.01 nM Sp-cAMPS stimulation, respectively. **(C)** Percentage (%) of cells displaying response to 0.001 nM,0.01 nM, 10 nM, and 10 μM Sp-cAMPS stimulation in WT and *c2gapA*^–^ cells, respectively. The same three independent experiments were used to calculate for **(C,D)**. Student’s *t*-test was used to calculate the *p*-value, which is indicated as *** (*p* < 0.001) and **** (*p* < 0.0001). **(D)** Percentage (%) of cells failing to display adaptation to 0.001, 0.01, 10 nM, and 10 μM cAMP stimulation in WT and *c2gapA*^–^ cells.

Little connection has been made between GPCR-mediated adaptation and the sensitivity of a cell. We noticed that C2GAP1 localized on the membrane of resting cells, suggesting its potential role in inhibiting the basal Ras activity of resting cells (Xu et al., 2017). It has also been previously shown that depletion of multiple PIP2 species, such as phosphatidylinositol-4,5-bisphosphate [PI(4,5)P2)] or phosphatidylinositol-3,4-bisphosphate [PI(3,4)P2)] increases Ras/Rap-related activities and excitability in a cell (Miao et al., 2017; Li et al., 2018). C2GAP1 binds to both PI(3,4)P2 and PI(4,5)P (Figure 2E). More importantly, cAMP-induced membrane translocation of C2GAP1 is F-actin independent (Xu et al., 2017). We speculated that the higher basal Ras activity in *c2gapA*^–^ cells might enhance their sensitivity to chemoattractant stimulation in an F-actin-independent fashion. Therefore, we next determined the sensitivity of both WT and *c2gapA*^–^ cells without the actin cytoskeleton (Figure 3A). Cells expressing the PIP_3_ biosensor PH_Crac_-GFP were treated with 5 μM Latrunculin B for 10 min prior to the experiment to diminish the existing cytoskeleton. In response to 10 μM Sp-cAMP stimulation, F-actin-free immobile WT cells displayed a transient membrane translocation of PH_Crac_-GFP (Supplementary Video 3, left), an adaptive behavior of WT cells. However, *c2gapA*^–^ cells displayed a secondary, persistent accumulation of PH_Crac_-GFP on the plasma membrane after its initial, transient membrane translocation, a non-adaptive behavior as previously shown (Supplementary Video 3, right) (Xu et al., 2017). In response to 0.1 nM Sp-cAMP stimulation, few WT cells (<15%) responded (Supplementary Video 4, left), while most of the *c2gapA*^–^ cells (>85%) showed clear membrane translocation of PH_Crac_-GFP (Supplementary Video 4, right). Quantitative measurement of the responsiveness and adaptation behavior of WT and *c2gapA*^–^ cells confirmed the above observation (Figures 3C,D). These results together demonstrate that *c2gapA*^–^ cells, lacking Ras inhibitor, are more sensitive to chemoattractant stimuli independent of the actin-based cytoskeleton.

### The Concentration Range of cAMP Gradients in Which Cells Chemotax Efficiently Is Upshifted in *c2gapA*^–^ Cells

**FIGURE 4:**
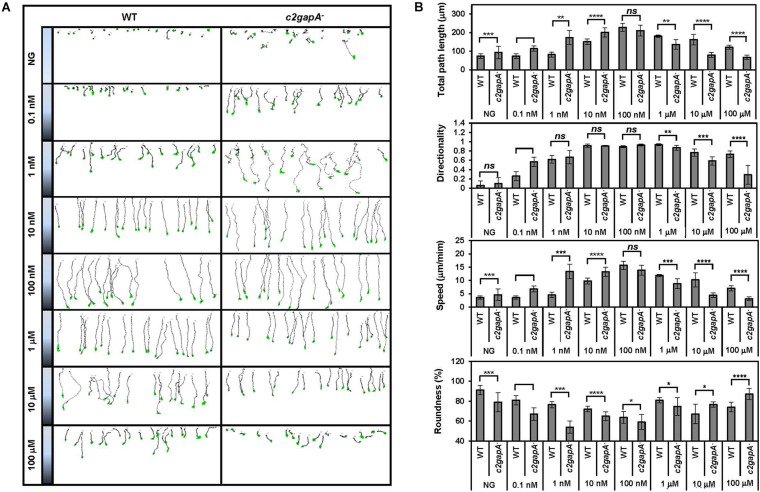
The concentration range of cAMP gradients in which cells chemotax efficiently is upshifted in *c2gapA*^–^ cells. **(A)** Montages show the traveling path of chemotaxing WT or *c2gapA*^–^ cells in response to gradients generated from a cAMP source at wide-range concentrations. Also see Supplementary Video 5. **(B)** Chemotaxis behaviors measured from **(A)** are described by four parameters: directionality, specifically “upward” directionality, where 0 represents random movement and 1 represents straight movement toward the gradient; speed, defined as the distance that the centroid of the cell moves as a function of time; total path length, the total distance the cell has traveled; and roundness (%) for polarization, which is calculated as the ratio of the width to the length of the cell. Thus, a circle (no polarization) is 1 and a line (perfect polarization) is 0. Twenty-five cells in each group were measured for 6.25 min. The mean ± SD is shown. The *p*-values of Student’s *t*-test are indicated as *ns* (not significant, *p* > 0.1), * (*p* < 0.1), ** (*p* < 0.01), *** (*p* < 0.001), or **** (*p* < 0.0001).

The basal activity of Ras is essential for the regulation of actin dynamics and, more importantly, promotes chemotaxis of *Dictyostelium* cells in a shallow chemoattractant gradient (van Haastert et al., 2017). We observed the localization of C2GAP1 not only on the protrusion sites but also on the plasma membrane of resting cells, indicating a role of C2GAP1 in regulating both basal and chemoattractant-stimulated Ras signaling (Xu et al., 2017). Consistent with the above, *c2gapA*^–^ cells displayed an enhanced basal Ras activity and sensitivity in response to chemoattractant stimulation (Figure 3). To precisely determine the function of C2GAP1 in chemotaxis in response to gradients at different concentrations, we monitored the chemotaxis behavior of WT and *c2gapA*^–^ cells in the gradients with a wide concentration range (Figure 4A). For the experiments, we used *EZ-TAXIScan* to apply a well-controlled linear gradient (Wen et al., 2016). Without a gradient, *c2gapA*^–^ cells displayed a bigger random walk than WT cells. In the gradients generated from a source concentration greater than 1 μM, *c2gapA*^–^ cells, in clear contrast to WT cells, displayed a significant decrease in the four parameters of chemotaxis: migration speed, directionality, total path length, and polarity (Figures 4A,B and Supplementary Video 5). When exposed to a gradient at a lower concentration (100 nM), WT and *c2gapA*^–^ cells displayed similar directionality. The above results are consistent with the previous report (Xu et al., 2017). Next, we monitored chemotaxis behaviors of both WT and *c2gapA*^–^ cells in response to gradients at low or subsensitive concentrations. We found that in the gradients generated from 1 and 10 nM cAMP sources, *c2gapA*^–^ cells displayed significantly improved chemotaxing behavior, especially in migration speed, directionality, and polarity, in comparison with CTL cells. In a subsensitive, 0.1 nM cAMP gradient, most WT cells (more than 70%) displayed random migration, while the majority of *c2gapA*^–^ cells displayed directional cell migration with improved directionality and migration speed. Taken together, these data indicate that *c2gapA*^–^ cells display defective chemotaxis in high-concentration gradients but improved chemotaxis in low- or subsensitive-concentration gradients. The above result indicates that WT cells chemotaxed efficiently through gradients of various chemoattractants ranging from 10*^–^*^9^ to 10*^–^*^6^ M, while *c2gapA*^–^ cells did so from 10*^–^*^10^ to 10*^–^*^7^ M. In other words, the concentration range of cAMP gradients in which cells chemotax efficiently is upshifted in *c2gapA*^–^ cells.

## Discussion

An amazing feature of eukaryotic cells is that they sense chemoattractants over an enormous concentration range. We have previously shown that C2GAP1 mediates cAMP receptor cAR1-mediated adaptation. Here, we reveal the molecular mechanism of C2GAP1 membrane targeting by which C2GAP1 controls both GPCR-mediated adaptation and the sensitivity of a cell. More importantly, we show that C2GAP1 allows *Dictyostelium* cells to sense chemoattractant at a higher concentration range through membrane targeting by lowering cell sensitivity and GPCR-mediated adaptation.

Calcium is involved in C2GAP1 membrane targeting. C2GAP1 has a calcium binding domain (C2 domain) that is often required for membrane targeting of the host proteins. In contrast to several other C2 domains, which require calcium binding for their membrane translocation (Damer and Creutz, 1994; Ilacqua et al., 2018), we found a decreased membrane localization of C2GAP1 in the presence of high [Ca^2+^] (Figure 1A). Consistent with the above result, *iplA*^–^ cells without [Ca^2+^] increase in response to cAMP stimulation, displayed an increased, prolonged membrane translocation of C2GAP1-YFP, while *cbpG*^–^ cells with higher basal [Ca^2+^] and stronger, quicker peaking [Ca^2+^] upon cAMP stimulation exhibited little membrane translocation of C2GAP1, instead, more further withdrawal of C2GAP1 from the plasma membrane to the cytoplasm in response to cAMP stimulation (Figures 1C–E; Wilczynska et al., 2005; Fisher and Wilczynska, 2006). An intracellular calcium gradient that was higher at the trailing edge of chemotaxing cells has been previously reported (Yumura et al., 1996). This spatial distribution of [Ca^2+^] might reinforce the leading-front targeting of C2GAP1 in the chemotaxing cells. However, the role of calcium in chemotaxis has been under debate for the past two decades. Initially, chemoattractant-induced calcium influx was thought to play no role in chemotaxis. This conclusion comes from the following two facts: firstly, mouse *plcb2*^–/^*^–^b3^–^*^/^*^–^* neutrophils without two major PLC isoforms, PLCβ2/β3, display impaired calcium influx yet show normal chemotaxis (Li et al., 2000); secondly, *Dictyostelium* cells lacking the IP_3_ receptor (*iplA*^–^) show no chemoattractant-induced Ca^2+^ response and chemotax just like WT cells do (Traynor et al., 2000). Recent studies demonstrate the essential role of chemoattractant-induced PLCβ/γ activation and subsequent Ca^2+^ response in neutrophil chemotaxis (Tang et al., 2011; Xu et al., 2015). More importantly, the chemotaxis assays in both studies in *Dictyostelium* gradients generated from 100 nM cAMP sources, in which we observed little difference in chemotaxis behavior between WT and *c2gapA*^–^ cells (Figure 3). Importantly, we observed more membrane localization of C2GAP1-YFP in resting *cbpG*^–^ cells that might be resulted from a higher basal [Ca^2+^] in the cells. Agreeing with the increased localization of C2GAP1 and subsequent downregulation of Ras/Rap1 function, *cbpG*^–^ cells display decreased adhesion and cell migration (Park et al., 2018). It is important to investigate the chemotaxis behavior of cells lacking mediators of calcium signaling, such as *iplA*^–^ and *cbpG*^–^ cells, in response to gradients of different concentrations.

Little connection has been made between GPCR-mediated adaptation and the basal activity of a cell. Interestingly, *Dictyostelium* cells lacking RasGAPs often display higher basal Ras activity (Zhang et al., 2008; Bloomfield et al., 2015; Xu et al., 2017), indicating that the regulation of basal Ras activity through RasGAPs might be a common mechanism in *Dictyostelium*. Recently, it has been shown that active Ras plays a role in cell locomotion and in chemotaxis through very shallow gradients (van Haastert et al., 2017). Thus, we examined the chemotaxis behavior of WT and *c2gapA*^–^ cells in response to a cAMP gradient over a large concentration range (10*^–^*^10^–10*^–^*^4^ M) (Figure 3) and found that in WT cells chemotaxed better than *c2gapA*^–^ cells did in gradients at high concentrations (>100 nM), while they displayed chemotaxis capability similar to that of *c2gapA*^–^ cells gradients at mid-concentration range (10 nM of cAMP). In gradients at low concentrations (<1 nM), *c2gapA*^–^ cells chemotaxed better than WT cells did, indicating a higher sensitivity of *c2gapA*^–^ cells. We further confirmed that the increased sensitivity of *c2gapA*^–^ cells is independent of F-actin (Figure 4). Importantly, human neutrophils lacking CAPRI, a RasGAP protein that controls Ras adaptation in GPCR-mediated chemotaxis, are more sensitive to chemoattractant gradients and sense gradients with a lower concentration range (Xuehua et al., 2020). In conclusion, membrane targeting of Ras inhibitors allows both human neutrophils and *D. discoideum* to sense chemoattractant gradients at a higher concentration range. These findings suggest that locally recruiting a Ras inhibitor of gradient sensing and chemotaxis for sensing an enormous concentration range of chemoattractants is an evolutionarily conserved mechanism.

## Materials and Methods

### Cell Lines, Cell Growth, and Differentiation

AX2 cells are the parent, WT cell line for *c2gapA*^–^ cells. *iplA*^–^ cells were from *Dictybase.org*. *cbpG*^–^ cells were from Park et al. (2018). Cells expressing the protein of interest were selected by growth in the presence of 20 μg/ml geneticin (Sigma, Steinheim, Germany) or 10 μg/ml blasticidin S, and/or hygromycin (Sigma, Steinheim, Germany) with the requirement of double selection. For differentiation, log-phase vegetative cells were harvested from shaking culture (5 × 10^6^ cells/ml) and washed twice with developmental buffer (DB: 5 mM Na_2_HPO_4_, 5 mM KH_2_PO_4_, 2 mM MgSO_4_, and 0.2 mM CaCl_2_). Cells were resuspended at 2 × 10^7^ cells/ml in shaking flask at 100 rpm, and allowed to differentiate with 75 nM adenosine 3′:5′-cyclic monophosphate (cAMP) (Sigma Aldrich, St. Louis, MO) pulses at 6 min intervals for 5–7 h or longer to obtain chemotactic cells. Differentiated cells were diluted to 1 × 10^7^ cells/ml in DB buffer with 2.5 mM caffeine and shaken at 200 rpm for 15 min.

### Plasmids

The YC-nano15 vector was obtained from Catherine Pears (Horikawa et al., 2010). PH_Crac_-RFP and C2GAP1-R616A-GFP were from AK, University of Groningen, Netherlands.

### Imaging and Data Processing

Differentiated cells (5 × 10^4^) in DB buffer with 2.5 mM caffeine were plated and allowed to adhere to the cover glass of a 4-well or a 1-well chamber (Nalge Nunc International, Naperville, IL) for 10 min, and then covered with DB buffer for the live cell imaging experiment. If necessary, cells were treated with 5.0 μM Latrunculin B (Molecular Probes, Eugene, OR) for 10 min prior to the experiments. Cells were imaged using a Carl Zeiss Laser Scanning Microscope Zen 780 (Carl Zeiss, Thornwood, NY) with a 40x/NA 1.4 Oil DIC Plan-Apochromatic objective. Images were processed and analyzed by Zen 780 software. Images were further processed in Adobe Photoshop (Adobe Systems, San Jose, CA), and the intensity of the ROI (region of interest) was explored and analyzed with Microsoft Office Excel (Redmond, WA). To measure the membrane translocation of the indicated protein, we first measured the intensity change of the cytoplasm in response to uniformly applied stimuli over time. To obtain the relative intensity change of each individual cell during the time lapse, we divided the cytosolic intensity at time 0 (I_0_) by its intensity at given time (I_*t*_); consequently, the relative intensity of any cells at time 0 became 1. To compensate for significant photobleaching that might occur with long-time acquisition, we also normalized the intensity relative to the photobleaching of the cells. Lastly, we calculated and presented the mean and standard deviation (Mean ± SD) of membrane translocation from more than five independent cells. To measure calcium response using the calcium sensor YC-nano15 with *D. discoideum cells* (Horikawa et al., 2010), we monitored FRET change by sensitized emission FRET measurement, a process previously reported in detail (Xu et al., 2016). The FRET signal change (Rt/R_0_) is presented as a function of [Ca^2+^] using Zen Software provided by Carl Zeiss Zen 780 software.

### Immunoprecipitation Assay

Cells were differentiated, washed with PM buffer (5 mM Na2PO4, 5 mM KH2PO4, and 2 mM MgSO4) with 2.5 mM caffeine, resuspended to 8 × 10^7^ cells per ml in same PM buffer, and kept on ice before assay. If necessary, the cells were stimulated with 10 μM cAMP. 0.5 ml aliquots of cells at indicated time points were lysed with 10 ml immunoprecipitation buffer (IB, 20 mM Tris, pH8.0, 20 mM MgCl_2_, 10% glycerol, 2 mM Na_3_VO_4_, 0.25% NP40, and complete 1 × EDTA-free proteinase inhibitor) for 30 min on ice. Cell extracts were centrifuged at 16,000 × *g* for 10 min at 4°C. Supernatant fractions were collected and incubated with 25 μl anti-GFP agarose beads at 4°C for 2 h. Beads were washed four times with immunoprecipitation buffer and proteins were eluted by boiling the beads in 50 μl SDS sample buffer. A second set of aliquots of the cells at each time point was taken for the assessment of total Ras protein in the sample. The indicated proteins were detected by Western blotting using specific antibodies.

### PIP Strip Assay

Cells were suspended with PM buffer and run through a filter system with 5 μm pores. The lysed cells were centrifuged at 16,000 rpm for one min. The supernatants were immediately mixed with immunoprecipitation buffer (IB, 20 mM Tris, pH8.0, 20 mM MgCl_2_, 10% glycerol, 2 mM Na_3_VO_4_, 0.25% NP40, and complete 1 × EDTA-free proteinase inhibitor) pre-cooled on ice. PIP-strip membranes were incubated with the mixtures obtained from the above at 4°C for 2 h and washed using IB buffer and then subject to western blot detection of the indicated proteins.

### *EZ-TAXIScan* Chemotaxis Assay and Data Analysis

The procedure was as previously reported (Wen et al., 2016). Briefly, differentiated cells were loaded onto one side of a 4-μm EZ-TAXIScan chamber. The chemoattractants at the indicated concentrations were added to the other side of the well across the terrace to generate a linear gradient which the cells chemotax through. The traveled distance is the traveled length (μm). The length of the terrace is the total length of the gradient generated (260 μm). The chemoattractant concentration of gradient (C) a cell experienced at each given position depends on both the concentration of the fMLP source (C_s__our__ce_) and the ratio of the distance the cell traveled from no gradient (the traveled length) to the total distance to the cAMP source (the total length). In other words, C = C_s__our__ce_ × ratio of the traveled length to the total length). The cells migrated for 30 min at room temperature. Images were taken for 30 min at 15 s intervals. For chemotaxis parameter measurements, 25 cells in each group were analyzed with DIAS software (Wessels et al., 1998). Chemotaxis behaviors are measured as four parameters: directionality, specifically “upward” directionality, where 0 represents random movement and 1 represents straight movement toward the gradient; speed, defined as the distance that the centroid of the cell moves as a function of time; total path length, which is the total distance the cell has traveled; and roundness (%) for polarization, which is calculated as the ratio of the width to the length of the cell. Thus, a circle (no polarization) is 1 and a line (perfect polarization) is 0. Student’s *t*-test was used to calculate the *p*-values. The bar graphs of chemotaxis parameters in mean ± SD were plotted with Microsoft Office Excel (Redmond, WA) or shown directly in the table.

## Data Availability Statement

The original contributions presented in the study are included in the article/Supplementary Material, further inquiries can be directed to the corresponding author/s.

## Author Contributions

XX designed the research. XX, SB, HP, and XW performed the research and analyzed the data. XX, AK, TJe, and TJi wrote the manuscript. All authors contributed to the article and approved the submitted version.

## Conflict of Interest

The authors declare that the research was conducted in the absence of any commercial or financial relationships that could be construed as a potential conflict of interest.

## Publisher’s Note

All claims expressed in this article are solely those of the authors and do not necessarily represent those of their affiliated organizations, or those of the publisher, the editors and the reviewers. Any product that may be evaluated in this article, or claim that may be made by its manufacturer, is not guaranteed or endorsed by the publisher.
