# Supplementary material for: Membrane Targeting of C2GAP1 Enables Dictyostelium discoideum to Sense Chemoattractant Gradient at a Higher Concentration Range
Source: Front Cell Dev Biol. 2021 Jul 30;9:725073. doi: 10.3389/fcell.2021.725073 (PMC8362602; doi:10.3389/fcell.2021.725073)
Supplement: Supplementary file 6 [file Data_Sheet_1.pdf]

Supplemental Information

Figure legends:

Figure S1. Membrane localization of C2GAP1 in WT, iplA-, and cbpG- cells.

A. Quantitative measurement of C2GAP1 on the plasma membrane and cytoplasm in WT, *iplA⁻*, and *cbpG⁻* cells is shown in left panel (right). The intensity of C2GAP1 (green) across a cell (left panel) was measured using Zen software and analyzed by Excel software.

B. Ratio of C2GAP1 intensity on the plasma membrane and the cytoplasm. Mean ± SD is shown; n = 3, 3, and 3, in WT, *iplA⁻*, and *cbpG⁻* cells, respectively. The *p* values of Student's t-test are indicated as *ns* (not significant, p > 0.1) and *** (p < 0.001).

Legends for supplemental videos

Videos S1. cAMP-induced membrane translocation of C2GAP1-YFP in wild-type (WT) and mutant cells. WT (top), *iplA⁻* (middle), or *cbpG⁻* (bottom) cells expressing C2GAP-YFP (green) without (left) or with 5 μM latrunculin treatment (right) treatment were stimulated with homogeneously applied 10 μM cAMP. To visualize the application of stimuli, cAMP was mixed with a fluorescent dye, Alexa 594 (red), and added to the cells at time 0 s. Scale bar and time are shown.

Video S2. cAMP stimulation triggers membrane translocation of WT or R616A mutant of C2GAP1 in the cells. Cells expressing GFP-tagged WT (left) or R616A mutant (right) were stimulated with homogeneously applied cAMP. To visualize the application of stimuli, cAMP was mixed with a fluorescent dye, Alexa 594 (red), and added to the cells at time 0 s. Scale bar and time are shown.

Video S3. 10 μM Sp-cAMP stimulation-induced PIP_3 production monitored by membrane translocation of PIP_3 biosensor PH_{Crac}-GFP (green) in WT (left) and *c2gapA*$^-$ (right) cells. To visualize the application of stimuli, cAMP was mixed with a fluorescent dye, Alexa 594 (red), and added to the cells at time 0 s. Scale bar and time are shown.

Video S4. 0.01 nM Sp-cAMP stimulation induced PIP_3 production monitored by membrane translocation of PIP_3 biosensor PH_{Crac}-GFP (green) in WT (left) and *c2gapA*$^-$ (right) cells. To visualize the application of stimuli, cAMP was mixed with a fluorescent dye, Alexa 594 (red), and added to the cells at time 0 s. Scale bar and time are shown.

Video S5. Chemotaxis of WT (left) and *c2gapA*$^-$ (right) cells experiencing gradients generated from dilution series of cAMP sources at the indicated concentrations using EZ-TAXIScan. cAMP gradients generated are linear. The concentration on the top side of the terrace is 0 and the concentrations on the bottom side of the terraces are as indicated on the left side of the terrace. The precise chemoattractant concentration (C) of gradient a cell experienced at a given position depends on the concentration of the fMLP source (C_{source}) and the ratio between the distance the cell traveled (the traveled length) from 0 concentration to the total distance (the total length) to the fMLP source. That is, $C = C_{source} \times$ ratio of traveled length vs total length.
